# Predictors of thyroglobulin in the lymph nodes recurrence of papillary thyroid carcinoma undergoing total thyroidectomy

**DOI:** 10.1186/s12893-021-01063-z

**Published:** 2021-01-22

**Authors:** Zhichao Xing, Yuxuan Qiu, Zhe Li, Lingyun Zhang, Yuan Fei, Jingqiang Zhu, Anping Su

**Affiliations:** 1 Center of Thyroid & Parathyroid Surgery, West China Hospital, Sichuan University, NO. 37 Guo Xue Xiang, Chengdu, Sichuan People’s Republic of China; 2 Department of Ultrasound, West China Hospital, Sichuan University, NO. 37 Guo Xue Xiang, Chengdu, China

**Keywords:** Thyroglobulin, Lymph nodes recurrence, Papillary thyroid carcinoma, Radioactive iodine treatment

## Abstract

**Background:**

To investigate the association between postoperative lymph nodes (LNs) recurrence and distinct serum thyroglobulin (Tg) levels in patients with papillary thyroid carcinoma (PTC).

**Methods:**

This study included PTC patients who underwent total thyroidectomy (TT) with at least central neck dissection and then re-operated due to recurrence of LNs between January 2013 and June 2018. These patients were grouped by negative or positive serum Tg levels according to the American Thyroid Association guidelines.

**Results:**

Of the 60 included patients, 49 underwent radioactive iodine (RAI) treatment. Maximum unstimulated Tg (uTg) ≥ 0.2 ng/mL were associated with larger diameter of recurrent LNs (*P* = 0.027), and higher rate of metastatic LNs (*P* < 0.001). Serum-stimulated Tg (off-Tg) ≥ 1 ng/mL (*P* = 0.047) and unstimulated Tg (on-Tg) ≥ 0.2 ng/Ml (*P* = 0.013) were associated with larger diameter of recurrent LNs. Number of metastatic LNs ≥ 8 was an independent predictor for postoperative maximum uTg ≥ 0.2 ng/mL (OR = 8.767; 95% CI = 1.392–55.216; *P* = 0.021). Ratio of metastatic LNs ≥ 25% was an independent predictor for off-Tg ≥ 1 ng/mL (OR = 20.997; 95% CI = 1.649–267.384; *P* = 0.019).

**Conclusion:**

Postoperative Tg-positive status was associated with larger size of recurrent LNs. Number of metastatic LNs ≥ 8 and ratio of metastatic LNs ≥ 25% were independent predicators for uTg-positive and off-Tg-positive status, respectively.

## Background

Papillary thyroid carcinoma (PTC) accounts for 85% of differentiated thyroid cancer and its incidence is rapidly growing worldwide [1]. PTC is often associated with lymph nodes (LNs) metastases in the central neck compartment (level VI) and then lateral neck compartment (level II to V) [2]. Total thyroidectomy (TT) with central neck dissection (CND) is often preferred as an initial surgical procedure for patients with thyroid cancer > 4 cm, or with gross extrathyroidal extension, or clinically visible metastatic disease to lymph nodes [1]. Selective or modified lateral neck dissection (LND) is performed under fine-needle aspiration cytology proven nodal metastasis [1]. Depending on the characteristics of the primary tumor and the risk of persistent or recurrent disease, postoperative radioactive iodine (RAI) can be necessary or recommended to prevent recurrence [1, 3]. Occasionally recurrences were still seen even if the surgery and subsequent RAI treatment were assumed, and the risk factors for these recurrences were N1b patients with age > 55 years or node metastasis > 3 cm [4].

Serum thyroglobulin (Tg) and anti-thyroglobulin antibody (TgAb) levels are recommended for assessing residual or recurrent diseases because the well-differential cancer or thyroid follicular cells are the only sources of serum Tg [1]. The serum Tg level is supposed to reach its lowest concentration at 3–4 weeks after TT. Therefore, Tg measurement from this point on is useful for detecting persistent or recurrent diseases [5]. Studies suggested the potential value of pre-RAI serum-stimulated Tg (off-Tg) on predicting recurrent diseases of metastatic PTC other than unstimulated Tg detected at 1 week after RAI ablation (on-Tg), although disputes still existed [6, 7]. At the same time, recurrence of LNs is the most common form of residual or recurrent diseases, apart from distant metastasis and biochemical recurrence, which is detectable under regular ultrasound examination during follow-up [8]. However, the relationship between serum Tg levels and the risk of recurrence of LNs remained unclear. Some studies demonstrated that Tg measurement could possibly serve as a useful negative predictor of persistent and recurrent PTC [9], while other authors reported serum Tg levels cannot be considered as reliable indicators for the absence of disease in patients already treated with RAI [10].

Thus, the aim of this study was to investigate the Tg levels in PTC patients who developed LNs recurrence, thereby evaluating probable risk factors and structural features of LNs recurrence to assist clinicians in selecting optimal therapy and postoperative surveillance.

## Methods

### Patients

Clinical database containing 901 consecutive records of patients with PTC in the Center of Thyroid & Parathyroid Surgery, West China Hospital, Sichuan University between January 2013 and June 2018 was reviewed. Patients with primary PTC who underwent TT and then re-operation due to LNs recurrence were included retrospectively. All surgeries were performed by one experienced surgeon team, and all recurrent LNs were confirmed by postoperative pathology. Tumors were staged according to the American Joint Committee for Cancer (AJCC) staging system (8th edition) [11]. Exclusive criteria included: (1) Patients underwent primary subtotal thyroidectomy or hemithyroidectomy and then recurred. (2) Patients did not undergo re-operations and no postoperative pathology were recorded. (3) Incomplete follow-up data. Proven or known thyroid remnant would be administered a therapeutic dose of radioactive iodine (RAI) dependent on outcomes of surgery in the next 1–2 months. Therefore, proven thyroid remnant was not exclusion criteria. This study was approved by the medical ethics committee of West China Hospital, Sichuan University.

### Treatments and follow-up

The primary surgery for included patients was TT with ipsilateral CND (level VI). Therapeutic CND would be performed in patients with pathologically involved LNs. If the prelaryngeal or pretracheal lymph nodes were confirmed metastatic by intraoperative frozen section, contralateral prophylactic CND would be performed. For patients with lateral LNs metastases diagnosed by preoperative fine needle aspiration or on an intraoperative frozen section, therapeutic LND (level II-V) would be conducted. After primary surgery, intermediate and high risk level PTC patients according to the American Thyroid Association (ATA) risk stratification system and patients with proven thyroid remnant would be administered a therapeutic dose of RAI dependent on outcomes of surgery in the next 1–2 months [1].

Off-Tg and on-Tg levels were tested in patients receiving RAI therapy. Neck ultrasound, serum-free thyroxine, thyroid stimulating hormone (TSH), maximum unstimulated Tg (uTg, did not include on-Tg) and TgAb were tested every 3 months the first year, every 6 months for the second year, and thereafter annually[12].

### Definitions

Off-Tg is defined as the Tg levels in condition of elevated TSH to > 70 IU/ml before RAI therapy and on-Tg is defined as the Tg levels in condition of thyroid hormone replacement after RAI therapy [1]. The maximum uTg < 0.2 ng/mL, on-Tg < 0.2 ng/mL and off-Tg < 1 ng/ml were deemed as negative Tg levels in the absence of interfering TgAb [1]. TgAb > 115 U/mL were considered positive TgAb levels in our center which could result in interference for Tg levels. All recurrent LNs were verified as structural recurrence with neck ultrasound. A fine needle aspiration was performed to confirm the recurrence and then a therapeutic neck dissection was performed in recurrent compartments.

### Groups and variants

Patients were divided into two groups according to their Tg status. Groups were first set up by maximum uTg levels by a cut-off level of 0.2 ng/mL. As for patients who were administered RAI therapy, they were divided into two groups by off-Tg with 1 ng/mL cut-off or by on-Tg with 0.2 ng/mL cut-off.

The following data were thoroughly reviewed: (1) Demographic data and basic information: age, sex, body mass index (BMI), nodular goiter (NG), hashimoto’s thyroiditis (HD), hypertension, diabetes, hyperthyroidism. (2) Surgical outcomes: surgical extent and associated harvested LNs (number of LNs removed during reoperation), metastatic LNs, metastatic rate of LNs, and diameters of recurrent LNs. (3) Oncological data: tumor size, multifocality, bilaterality, TNM stage, extranodal extension.

### Statistical analysis

Continuous variables were expressed as mean ± standard deviation (SD). The *χ*^2^ test or Fisher exact test was used to evaluate the differences of incidences, and the student’s *t*-test and/or analysis of variance was used to evaluate the differences of continuous variables. Based on the variables that were statistically significant or *P* value < 0.25 in univariate analysis, multivariate analysis with logistic regression was conducted to identify the independent risk factors. The variable was re-coded from a continuous variable into a dichotomous variable according to the optimal cut-off value produced by the receiver operator characteristic (ROC) curve analysis for multivariate analysis. The results of the multivariate analysis were reported as odds ratio (OR) with 95% confidence interval (CI). Two-sided *P* < 0.05 was considered statistically significant. All statistical analyses were performed using IBM SPSS Statistics version 25.0 for Windows (IBM Corp., Armonk, NY).

## Results

### Patient characteristics

**Fig. 1:**
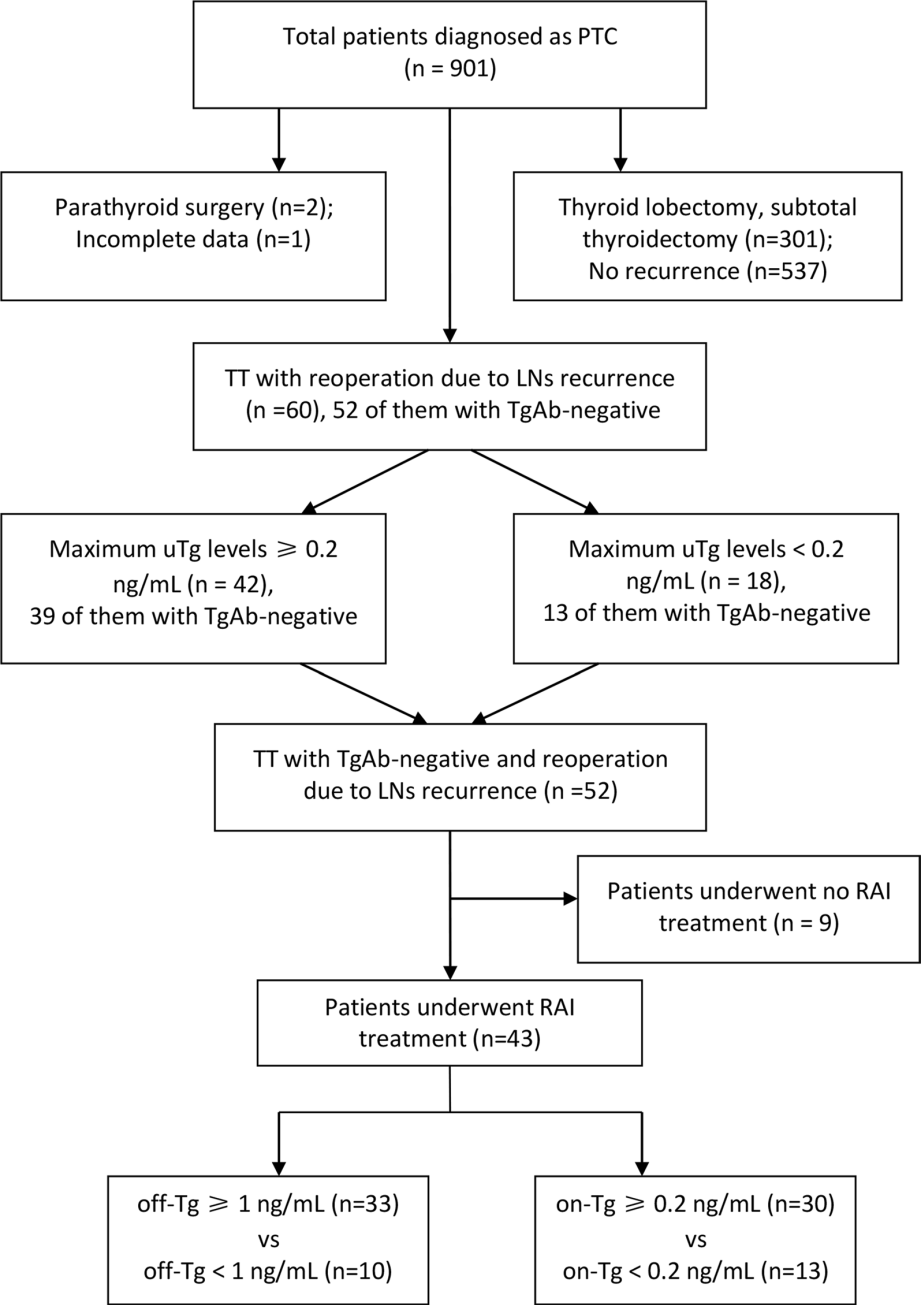
Flow chart of patients reviewed. *PTC* papillary thyroid carcinoma, *TT* total thyroidectomy, *LNs* lymph nodes, *Tg* thyroglobulin, *TgAb* anti-thyroglobulin antibody, *uTg* maximum unstimulated Tg, *RAI* radioactive iodine, *off-Tg* Pre-RAI ablation serum-stimulated Tg, *on-Tg* unstimulated Tg detected at 1 week after RAI ablation

Sixty patients (18 males and 42 females) were included and the flow chart was displayed in Fig. 1. The mean age was 44.1 ± 13.8 years (interquartile range, IQR, 33–54 years) and age ≥ 45 and ≥ 55 were seen in 29 (48.3%) and 14 (23.3%) patients, respectively. Eight (13.3%) patients were considered as TgAb-positive (TgAb > 115U/mL). Forty-nine (81.7%) patients underwent RAI therapy. The characteristics of the patients were presented in Table 1. The mean size of the tumor was 21.7 ± 14.3 mm and tumor diameter > 40 mm were found in 7 (11.7%) patients. Multifocality and bilaterality were identified in 11 (18.3%) and 8 (13.3%) patients, respectively. T3 stage was found in 16 (26.7%) patients while T4 in 19 (31.7%) patients. The mean numbers of total harvested and involved LNs were 33.0 ± 20.8 and 8.5 ± 8.2, respectively.

**Table 1:** Characteristics of the patients (N = 60)

Variable	N	%
Ages, yrs, mean ± SD (IQR)	44.1 ± 13.8 (33–54)	–
≥ 45	29	48.3
≥ 55	14	23.3
Sex, male/female	18/42	30.0/70.0
BMI	23.1 ± 3.7	
NG	24	40.0
HD	8	13.3
Hypertension	5	8.3
Diabetes	3	5.0
Hyperthyroidism	5	8.3
Hypothyroidism	0	0
Tumor size (primary), mm		
Mean ± SD (IQR)	21.7 ± 14.3 (10.75–22.25)	
> 40 mm	7	11.7
Multifocality (primary)	11	18.3
Bilaterality (primary)	8	13.3
TgAb positive (> 115U/mL)	8	13.3
TNM stage (primary)		
Tx/T1/T2/T3/T4	8/11/6/16/19	13.3/18.3/10.0/26.7/31.7
Nx/N0/N1a/N1b	0/11/12/37	0/18.3/20.0/61.7
Mx/M0/M1	1/59/0	1.7/98.3/0
Surgical strategy		
iCND/bCND	4/15	6.7/25.0
iCND + iLND	7	11.7
bCND + iLND	24	40.0
bCND + bLND	10	16.7
Harvested LNs	33.0 ± 20.8	
Metastatic LNs	8.5 ± 8.2	
The ratio of metastatic LNs %	29.5 ± 23.1	
RAI Administration	49	81.7
Central LNs recurrence	19	31.7
Lateral LNs recurrence	36	60.0
Central & lateral LNs recurrence	5	8.3
Diameters of recurrent LNs, mm, Mean ± SD (IQR)	18.7 ± 12.9 (10.75–22.00)	
Extranodal extension	20	33.3

*BMI* Body Mass Index, *HD* Hashimoto's disease, *NG* nodular goiter, *iCND* ipsilateral central neck dissection, *bCND* bilateral central neck dissection, *iLND* ipsilateral lateral neck dissection, *bLND* bilateral lateral neck dissection, *LN* lymph node

### Follow-up

During the follow-up with a median of 29 months (range, 13–78 months), central, lateral and both compartments LN recurrence were noted in 19 (31.7%), 36 (60.0%) and 5 (8.3%) patients, respectively. The mean diameter of the largest recurrent LN was 18.7 ± 12.9 mm. Extranodal extension was observed in 20 (33.3%) patients. All patients stayed alive till the end of follow-up.

Maximum uTg levels ≥ 0.2 ng/mL were detected in 42 (70%) patients and maximum uTg levels < 0.2 ng/mL were found in 18 (30%) patients during the follow-up. In 49 patients who underwent RAI treatment, 34 (69.4%) had an off-Tg ≥ 1 ng/mL and 15 (30.6%) had an off-Tg < 1 ng/mL, while on-Tg ≥ 0.2 ng/mL and on-Tg < 0.2 ng/mL were observed in 31 (63.3%) and 18 (36.7%) patients, respectively.

Meanwhile, in 52 patients with TgAb-negative (< 115 U/mL), 39 (75.0%) and 13 (25.0%) patients were appeared with maximum uTg levels ≥ 0.2 ng/mL and < 0.2 ng/mL respectively (Table 2). Forty-three patients received RAI and 33 (76.7%) of them had an off-Tg ≥ 1 ng/mL, and 10 (23.3%) of them had an off-Tg < 1 ng/mL (Table 3), while 30 (69.8%) had an on-Tg ≥ 0.2 ng/mL and 13 (30.2%) had an on-Tg < 0.2 ng/mL (Table 4).

**Table 2:** Comparisons of maximum uTg with TgAb-negative

	uTg ≥ 0.2 ng/ml (N = 39)	uTg < 0.2 ng/ml (N = 13)	t/χ^2^	P
Age, yrs	47.2 ± 14.1	39.2 ± 12.1	− 1.827	0.074
Sex (male)	15	3	0.453	0.501
BMI	23.7 ± 3.9	22.5 ± 3.8	− 0.938	0.353
NG	16	6	0.105	0.746
HD	5	1	< 0.001	1.000
Hypertension	4	2	< 0.001	1.000
Diabetes	2	1	–	1.000
Hyperthyroidism	3	2	0.074	0.786
Tumor size (primary), mm	21.7 ± 13.3	18.9 ± 10.4	− 0.682	0.498
Multifocality (primary)	4	5	3.628	0.057
Bilatrality (primary)	4	2	< 0.001	1.000
Surgical strategy				
iCND	2	1	–	1.000
bCND	11	4	–	1.000
bCND + iLND	20	8	–	0.403
bCND + bLND	8	1	–	0.403
Harvested LNs	32.7 ± 21.6	40.5 ± 19.7	1.148	0.256
Metastatic LNs	9.6 ± 8.8	5.9 ± 7.3	− 1.356	0.181
The ratio of metastatic LNs %	34.1 ± 25.7	14.4 ± 10.5	− 3.900	0.001*
Diameter of recurrent LNs	21.1 ± 14.7	14.6 ± 5.6	− 2.279	0.027*
≥ 25 mm	13	0	5.778	0.023*
Extranodal extension	15	5	< 0.001	1.000

*BMI* Body Mass Index, *HD* Hashimoto's disease, *NG* nodular goiter, *iCND* ipsilateral central neck dissection, *bCND* bilateral central neck dissection, *iLND* ipsilateral lateral neck dissection, *bLND* bilateral lateral neck dissection, *LNs* lymph nodes

*Means significantly statistical differences

**Table 3:** Comparisons of Off-Tg with TgAb-negative and RAI administration

	Off-Tg ≥ 1 ng/ml (N = 33)	Off-Tg < 1 ng/ml (N = 10)	t/χ^2^	*P*
Age, yrs	46.2 ± 14.3	38.4 ± 16.0	− 1.478	0.147
Sex (male)	13	3	0.027	0.869
BMI	23.4 ± 3.5	22.5 ± 3.3	− 0.776	0.442
NG	16	3	0.446	0.504
HD	4	1	< 0.001	1.000
Hypertension	3	1	–	1.000
Diabetes	1	1	–	0.415
Hyperthyroidism	0	2	–	1.000
Tumor size (primary), mm	22.7 ± 14.0	18.7 ± 11.0	− 0.829	0.412
Multifocality (primary)	3	4	3.351	0.067
Bilatrality (primary)	3	1	–	1.000
Surgical strategy				
iCND	0	0	–	–
bCND	7	2	–	–
bCND + iLND	19	7	–	0.645
bCND + bLND	7	1	–	0.645
Harvested LNs	37.6 ± 20.8	38.2 ± 21.8	0.074	0.941
Metastatic LNs	10.3 ± 9.2	6.8 ± 8.2	− 1.079	0.287
The ratio of metastatic LNs %	29.8 ± 23.6	16.3 ± 10.7	− 1.744	0.089
Diameter of recurrent LNs	23.0 ± 15.0	15.6 ± 4.7	− 2.472	0.018*
≥ 25 mm	13	0	3.933	0.047*
Extranodal extension	11	6	1.304	0.254

*BMI* Body Mass Index, *HD* Hashimoto's disease, *NG* nodular goiter, *iCND* ipsilateral central neck dissection, *bCND* bilateral central neck dissection, *iLND* ipsilateral lateral neck dissection, *bLND* bilateral lateral neck dissection, *LNs* lymph nodes

*Means significantly statistical differences

**Table 4:** Comparisons of on-Tg with TgAb-negative and RAI administration

	On-Tg ≥ 0.2 ng/ml (N = 30)	On-Tg < 0.2 ng/ml (N = 13)	t/χ^2^	*P*
Age, yrs	47.2 ± 14.5	38.0 ± 14.2	− 1.917	0.062
Sex (male)	12	4	0.054	0.817
BMI	23.4 ± 3.5	22.9 ± 3.3	− 0.417	0.679
NG	13	6	0.029	0.864
HD	4	1	< 0.001	0.990
Hypertension	3	1	< 0.001	1.000
Diabetes	1	1	–	0.518
Hyperthyroidism	2	0	–	1.000
Tumor size (primary), mm	22.6 ± 14.0	19.9.0 ± 11.8	− 0.594	0.556
Multifocality (primary)	2	5	4.597	0.032*
Bilatrality (primary)	2	2	0.110	0.740
Surgical strategy				
iCND	0	0	–	–
bCND	6	3	–	–
bCND + iLND	17	9	–	0.385
bCND + bLND	7	1	–	0.385
Harvested LNs	38.4 ± 20.5	36.3 ± 22.3	− 0.300	0.766
Metastatic LNs	10.9 ± 9.4	6.2 ± 7.4	− 1.588	0.120
The ratio of metastatic LNs %	29.9 ± 24.0	19.1 ± 14.4	− 1.500	0.141
Diameter of recurrent LNs	23.6 ± 15.5	16.0 ± 4.7	− 2.438	0.020*
≥ 25 mm	13	0	6.151	0.013*
Extranodal extension	11	6	0.341	0.559

*BMI* Body Mass Index, *HD* Hashimoto's disease, *NG* nodular goiter, *iCND* ipsilateral central neck dissection, *bCND* bilateral central neck dissection, *iLND* ipsilateral lateral neck dissection, *bLND* bilateral lateral neck dissection, *LNs* lymph nodes

*Means significantly statistical differences

### Univariate analyses of factors associated with positive Tg levels

The maximum uTg ≥ 0.2 ng/mL were significantly associated with older age (*P* = 0.024), higher rate of diameters of recurrent LNs ≥ 25 mm (*P* = 0.045) and higher LNs metastatic rate (*P* = 0.039), respectively. In 49 patients underwent RAI treatment, off-Tg ≥ 1 ng/mL were significantly associated with older age (*P* = 0.042), larger diameter of recurrent LNs (*P* = 0.021) and higher rate of diameters of recurrent LNs ≥ 25 mm (*P* = 0.038), respectively. Older age (*P* = 0.017), larger diameter of recurrent LNs (*P* = 0.020) and higher rate of diameters of recurrent LNs ≥ 25 mm (*P* = 0.008) were also seen in patients with on-Tg ≥ 0.2 ng/ml. However, lower rate of multifocality of the primary tumor was found in patients with off-Tg ≥ 1 ng/mL (*P* = 0.047) and on-Tg ≥ 0.2 ng/ml (*P* = 0.039), respectively.

When excluding TgAb-positive patients, the maximum uTg levels ≥ 0.2 ng/ml were significantly associated with larger diameter of recurrent LNs (P = 0.027), higher rate of diameters of recurrent LNs ≥ 25 mm (*P* = 0.023) and higher LN metastatic rate (*P* < 0.001; Table 2). In the 43 patients with TgAb-negative who underwent RAI treatment, off-Tg ≥ 1 ng/mL was significantly associated with larger diameter of recurrent LNs (*P* = 0.018) and higher rate of diameters of recurrent LNs ≥ 25 mm (*P* = 0.047; Table 3). Larger diameter of recurrent LNs (*P* = 0.020), higher rate of diameters of recurrent LNs ≥ 25 mm (*P* = 0.013), and lower rate of multifocality (*P* = 0.032) were also seen in patients with on-Tg ≥ 0.2 ng/mL (Table 4).

### Independent predictors of positive Tg levels

In multivariate analysis, the number of metastatic LNs ≥ 8 was an independent predictor for maximum uTg ≥ 0.2 ng/mL in patients with TgAb-negative (OR = 8.767; 95% CI = 1.392–55.216; *P* = 0.021), while multifocality was an independent protective factor for maximum uTg ≥ 0.2 ng/mL (OR = 0.123; 95% CI = 0.020–0.762; *P* = 0.024). As for patients received RAI with TgAb-negative, the ratio of metastatic LNs ≥ 25% was an independent predictor for off-Tg ≥ 1 ng/mL (OR = 20.997; 95% CI = 1.649–267.384; *P* = 0.019). However, no significant differences were found in the multivariate analysis for predictors of on-Tg ≥ 0.2 ng/mL (Table 5).

**Table 5:** Multivariate analysis of predictors of maximum uTg ≥ 0.2 ng/mL with TgAb-negative

	*P*	OR	95% CI
Multifocality	0.024*	0.123	0.020–0.762
Number of metastatic LNs ≥ 8	0.021*	8.767	1.392–55.216

*OR* odds ratio, *CI* confidence interval, *LNs* lymph nodes

*Means significantly statistical differences

## Discussion

We included PTC patients with recurrence of LNs and tried to find out the differences between Tg-negative or positive during the follow-up. Our current study cohort was well followed with a median period of 29 months but subjected by a notable sample size (60 patients). Though many PTC patients underwent secondary surgery for recurrence in our center, the primary treatment of partial them were performed elsewhere and it became difficult to obtain the whole and correct data. In order to ensure the integrality and avoid the bias coming from incomplete outcomes, we decided to establish strict inclusive criteria other than simply enlarging the sample size. It was recognized that positive TgAb levels would affect the serum Tg levels and some studies excluded all patients with TgAb-positive to avoid potential interference [7, 13]. The recent study concluded that TgAb interference limits Tg utility as a tumor marker in 30% of TgAb-positive patients [14]. However, we did not exclude patients with TgAb-positive in our study because we found the serum Tg levels of the patients with TgAb-positive were far from the threshold and the interference of TgAb levels would not affect the results of the grouping. Nevertheless, to ensure the most preciseness, subgroup excluding patients with TgAb-positive was still conducted.

Our results indicated that patients with maximum uTg ≥ 0.2 ng/mL were significantly associated with older age, diameters of recurrent LNs ≥ 25 mm and higher LNs metastatic rate at primary surgery. Age is often associated with tumor malignancy and 55 years old is regarded as a cut-off age in the 8^th^ edition AJCC staging system [11]. However, age was no longer an independent predictor when we excluded patients with TgAb-positive. Higher metastatic rates seen in the maximum uTg ≥ 0.2 ng/mL group further indicated tumor malignancy and invasiveness.

Positive off-Tg levels and positive on-Tg levels were significantly associated with a larger diameter of recurrent LNs. The sources of Tg were recurrent LNs and the levels of Tg theoretically depended on numbers of recurrent LNs and size of each recurrent LNs when there were no other remnant areas, and the levels of Tg were also regulated by TSH levels. No difference of numbers of metastatic LNs and TSH levels was found when performing the analysis of positive and negative groups of off-Tg or on-Tg. This similar trend was observed when comparing maximum uTg-positive group and maximum uTg-negative group in all included patients. This might be due to the large proportion of RAI patients (81.7%) which add to this trend and possibly existed remnant thyroid or tumor tissues in patients without RAI administration shared the source which finally led to no difference. RAI administration was considered the effective way to ablate remnant thyroid tissue though incomplete structural response sometimes occurred [15].

Off-Tg testing is recommended to obtain the best follow-up accuracy for recurrent or persistent disease detection than on-Tg [16]. In the multivariate analysis, a ratio of metastatic LNs ≥ 25% was the only independent predicator for off-Tg ≥ 1 ng/mL, whereas no predicator was found in on-Tg ≥ 0.2 ng/mL. A recent study showed an initial off-Tg level of 5.0 ng/mL was involved in predicting recurrence with the highest sensitivity and specificity [7]. This is consistent with the results of a previous study, which defined the cut-off point for positive or negative value at 4.2 ng/mL [17]. However, off-Tg < 1 ng/mL was considered negative in our analysis for a recent study suggested that off-Tg levels were correlated with structural incomplete response: 0% structural incomplete response with the off-Tg levels of < 1 ng/mL, 1.73% with the off-Tg levels of 1–10 ng/mL, and 42.74% with the off-Tg levels of > 10 ng/mL, which corresponded to the guideline (2015) [1, 6]. Orlov et al. proposed that patients with an off-Tg < 1 ng/mL should not receive RAI and those with off-Tg 1–5 ng/mL should be further evaluated based on repeat off-Tg levels and pathologic features [18]. However, the proportion (10/43, 23.3%) of patients with off-Tg negative (< 1 ng/mL) was not low as expected in patients with LNs recurrence. Thus, we thought the cut-off value of off-Tg in patients with risk of structural recurrence should be further discussed. Meanwhile, number of metastatic LNs ≥ 8 showed an independent indicator for maximum uTg ≥ 0.2 ng/mL. Alexandria et al. similarly proposed a strategy to use maximum uTg levels for clinical decision making rather than off-Tg levels [15]. Patients with maximum uTg ≥ 0.2 ng/mL did benefit from RAI administration as it effectively ablates remnant thyroid tissue, which might allow for easier biomedical detection of recurrence [15]. Our results suggested patients with maximum uTg < 0.2 ng/mL have already achieved an excellent response from surgery with similar number of harvested LNs and lower ratio of metastatic LNs but still required attention on imaging during follow-up.

Our analysis did have limitations inherent in its study design. The data were retrospectively collected. In addition, a lot of patients were excluded for incomplete data of Tg and primary surgery, thus leading to the not notable sample size. We only focused on LNs recurrence, and other types of recurrence such as distant recurrence and biochemical recurrence were not analyzed. The accurate time of the recurrent was difficult to decide.

## Conclusions

This study found Tg-positive was associated with larger size of recurrent LNs. Number of metastatic LNs ≥ 8 could independently predict maximum uTg-positive with negative TgAb levels. The ratio of metastatic LNs ≥ 25% was an independent predictor for off-Tg-positive in patients with negative TgAb levels and RAI administration. RAI administration seems to benefit the value of Tg measurement during follow-up. However, importantly, imaging examinations need to be further applied for Tg-negative patients on account of difficulty on detecting Tg with smaller LNs, especially for patients without RAI ablation.

## Data Availability

Datasets from the current study are available from the corresponding author on reasonable request.

## Abbreviations

Tg: Serum thyroglobulin
PTC: Papillary thyroid carcinoma
LNs: Lymph nodes
TT: Total thyroidectomy
RAI: Radioactive iodine
uTg: Unstimulated Tg
off-Tg: Pre-RAI ablation serum-stimulated Tg
on-Tg: Unstimulated Tg detected at 1 week after RAI ablation
CND: Central neck dissection
LND: Lateral neck dissection
TgAb: Anti-thyroglobulin antibody
TSH: Thyroid stimulating hormone
BMI: Body mass index
NG: Nodular goiter
HD: Hashimoto’s thyroiditis
ROC: Receiver operator characteristic
OR: Odds ratio
CI: Confidence interval
